# Supplementary material for: QTL analysis of femaleness in monoecious spinach and fine mapping of a major QTL using an updated version of chromosome-scale pseudomolecules
Source: PLoS One. 2024 Feb 23;19(2):e0296675. doi: 10.1371/journal.pone.0296675 (PMC10890751; doi:10.1371/journal.pone.0296675)
Supplement: S1 Fig — (PDF) [file pone.0296675.s001.pdf]

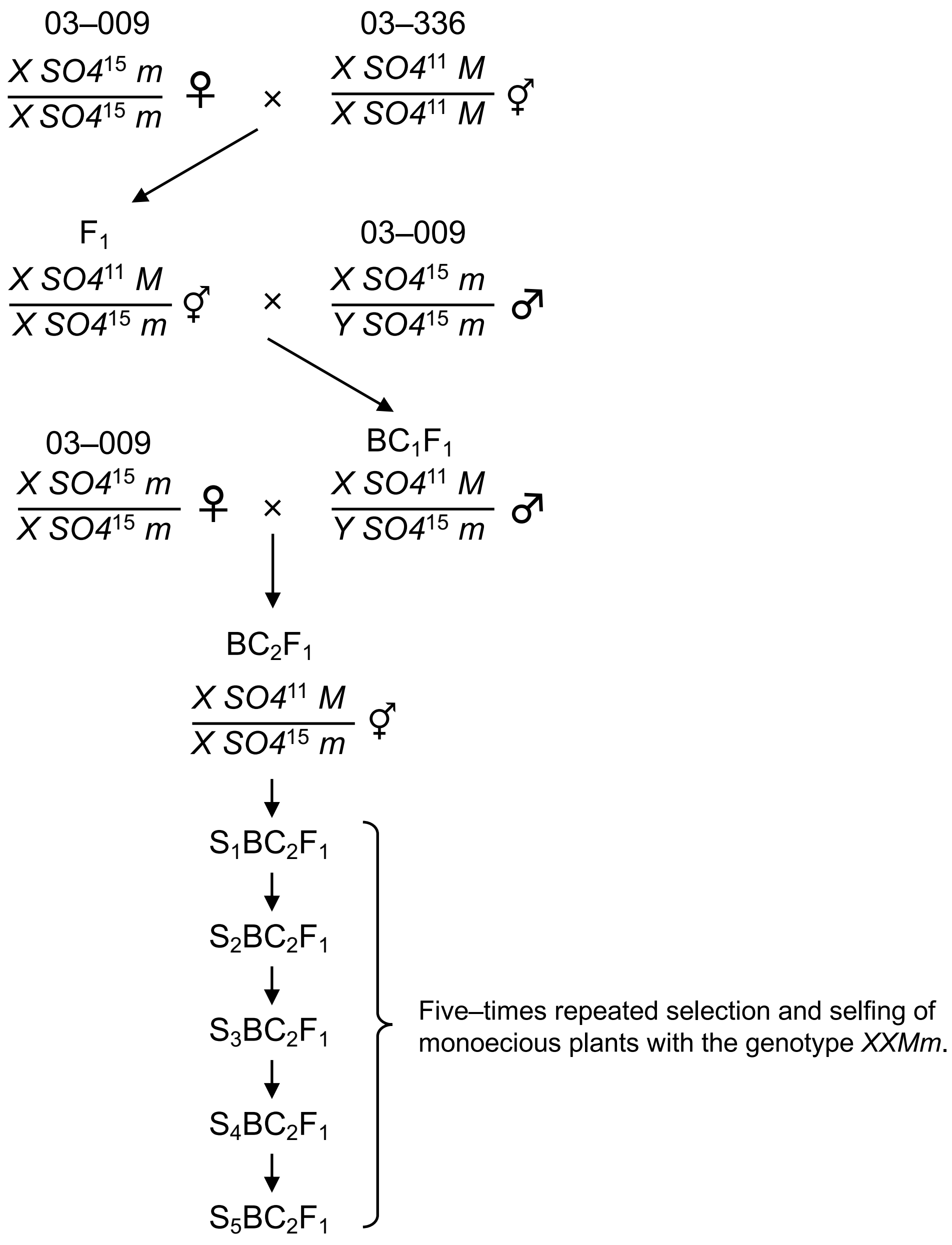

S1 Fig. Crossing scheme to produce self–pollinated progeny families from a monoecious selection in a 03–009 × 03–336 BC_2F_1 population.
