# Supplementary figures and images for: QTL analysis of femaleness in monoecious spinach and fine mapping of a major QTL using an updated version of chromosome-scale pseudomolecules
Source: PLoS One. 2024 Feb 23;19(2):e0296675. doi: 10.1371/journal.pone.0296675 (PMC10890751; doi:10.1371/journal.pone.0296675)

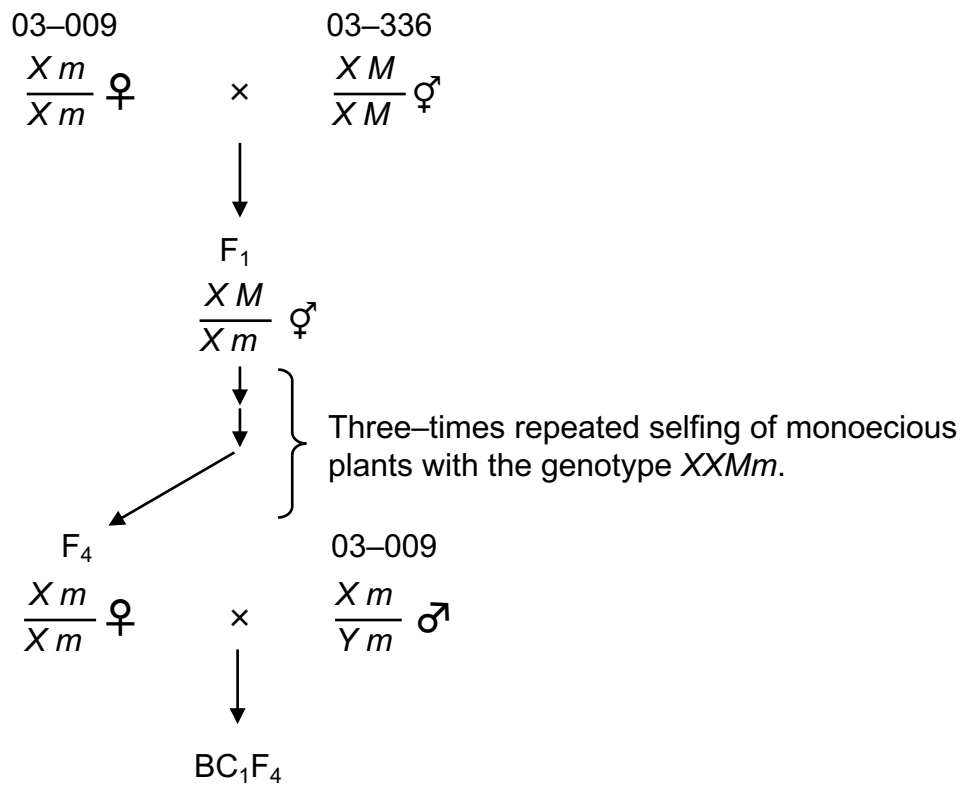

**S2 Fig. Crossing scheme to produce 03-009 × 03-336 BC<sub>1</sub>F<sub>4</sub>.**

Supplement: S2 Fig — (PDF) [file pone.0296675.s002.pdf]

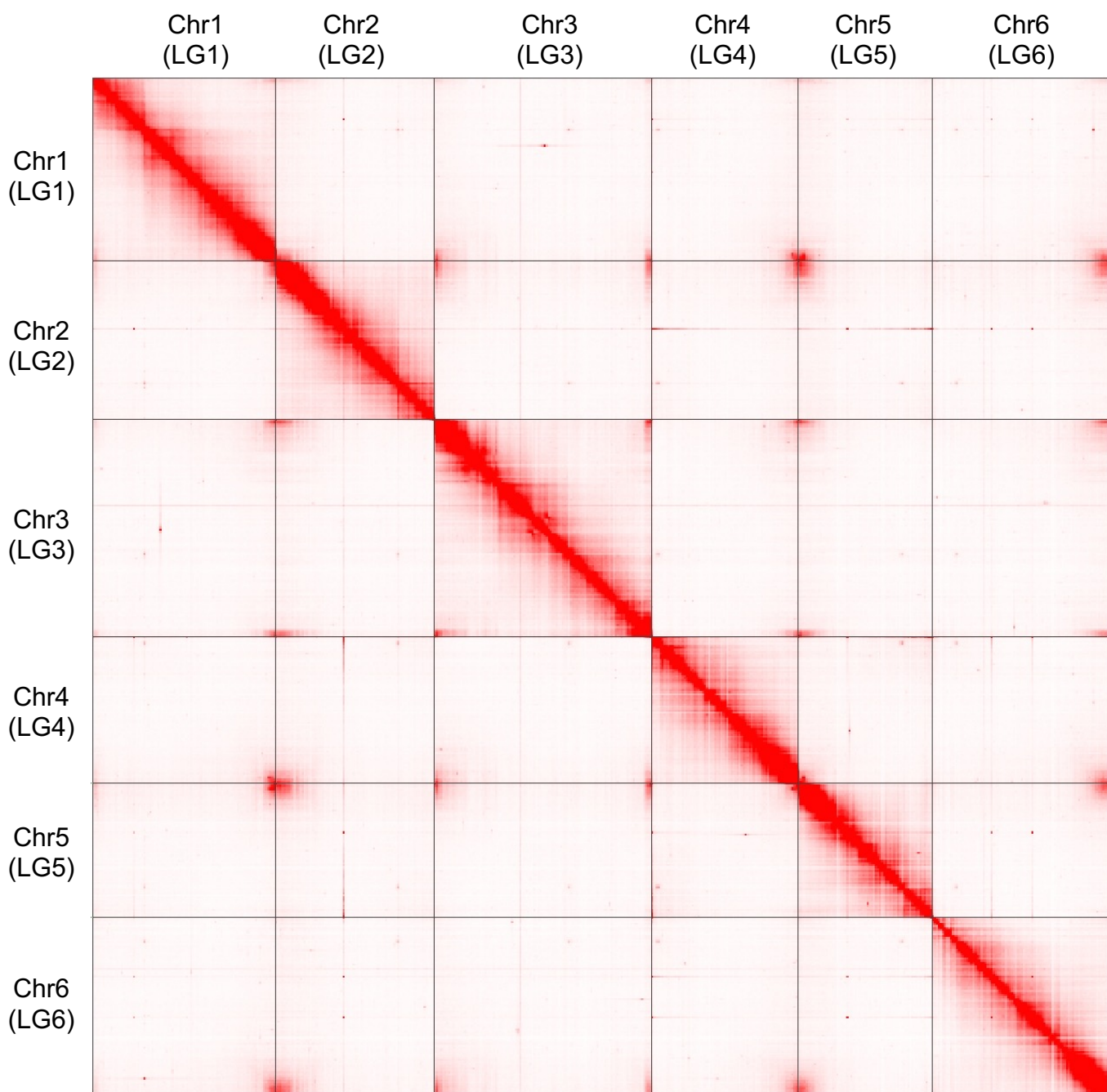

**S5 Fig. Hi-C contact map of the chromosomal pseudomolecules SOL\_r2.0\_pseudomolecule.**

Supplement: S5 Fig — (PDF) [file pone.0296675.s005.pdf]
