# Supplementary material for: QTL analysis of femaleness in monoecious spinach and fine mapping of a major QTL using an updated version of chromosome-scale pseudomolecules
Source: PLoS One. 2024 Feb 23;19(2):e0296675. doi: 10.1371/journal.pone.0296675 (PMC10890751; doi:10.1371/journal.pone.0296675)
Supplement: S3 Fig — (PDF) [file pone.0296675.s003.pdf]

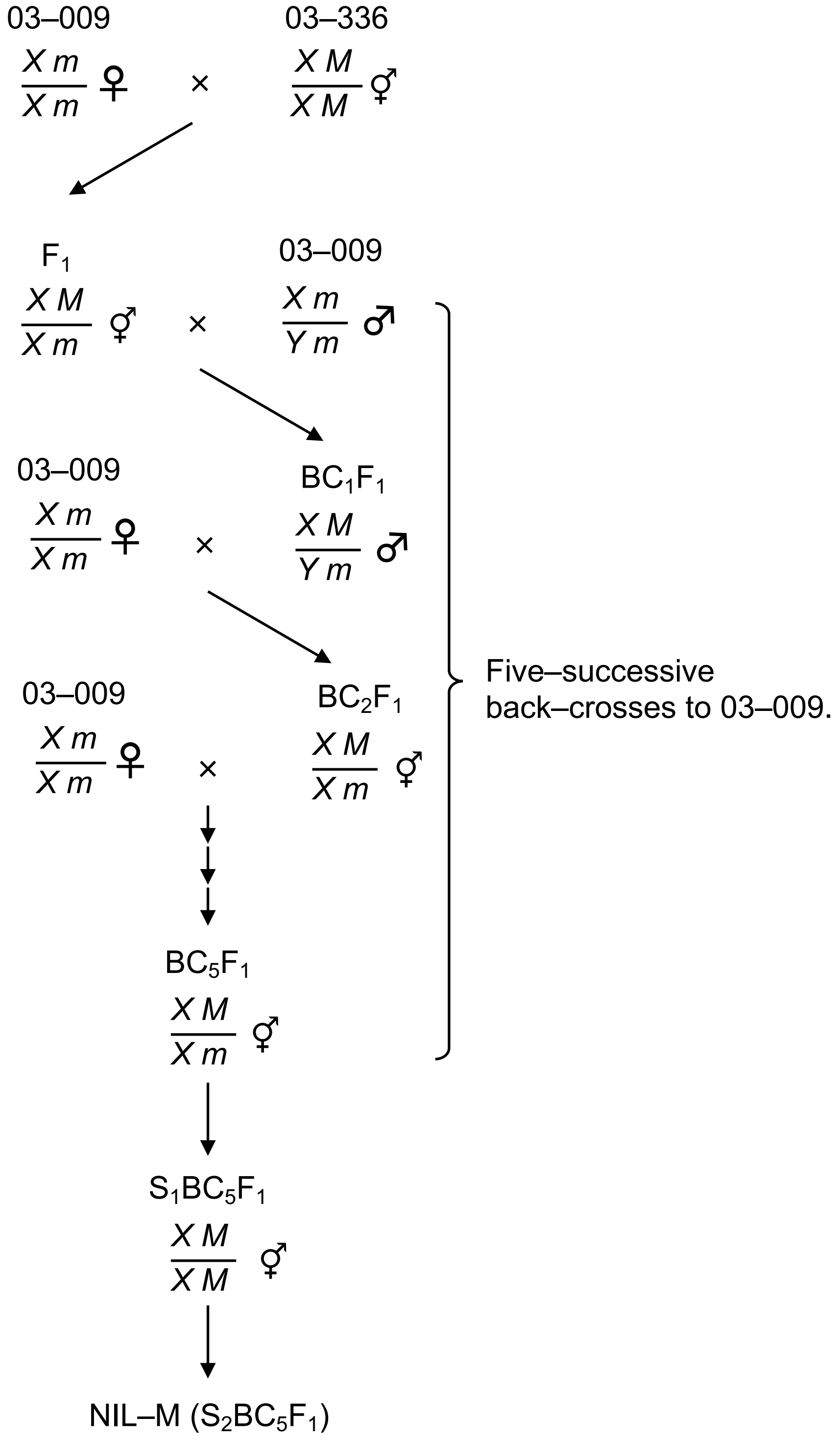

S3 Fig. Crossing scheme to obtain the line NIL–M bearing the *XXMM* genotype in the background of the dioecious line 03–009.
