# Supplementary material for: QTL analysis of femaleness in monoecious spinach and fine mapping of a major QTL using an updated version of chromosome-scale pseudomolecules
Source: PLoS One. 2024 Feb 23;19(2):e0296675. doi: 10.1371/journal.pone.0296675 (PMC10890751; doi:10.1371/journal.pone.0296675)
Supplement: S4 Fig — (PDF) [file pone.0296675.s004.pdf]

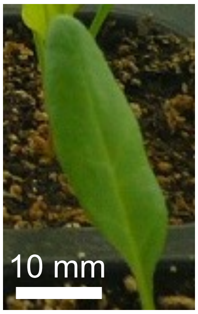

A leaf from a NIL-M plant under short-day conditions

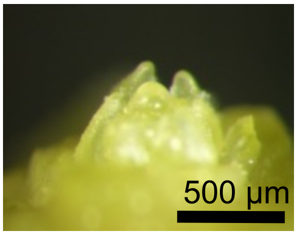

A shoot apex in a NIL-M plant under short-day conditions

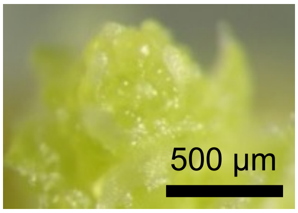

shoot apexes in 3-days long day exposed plants

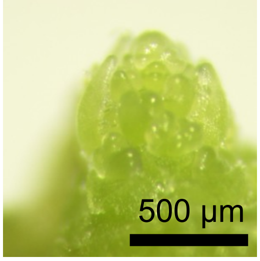

A shoot apex in a 7-days long day exposed NIL-M plant

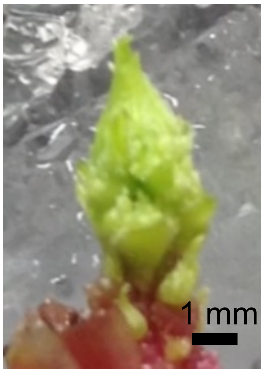

Early-stage inflorescence from a NIL-M plant

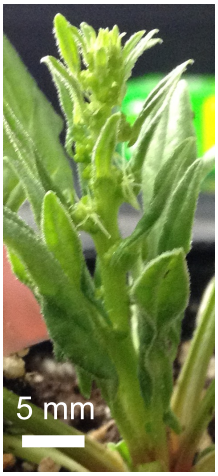

Mid-stage inflorescence from a NIL-M plant

S4 Fig. Representative images of plant tissues used for RNA extraction.
