# Supplementary material for: QTL analysis of femaleness in monoecious spinach and fine mapping of a major QTL using an updated version of chromosome-scale pseudomolecules
Source: PLoS One. 2024 Feb 23;19(2):e0296675. doi: 10.1371/journal.pone.0296675 (PMC10890751; doi:10.1371/journal.pone.0296675)
Supplement: S6 Fig — a. A schematic diagram showing the physical positions of qFem3.1 and DNA markers on a pseudomolecule, Chr3. b. Graphical genotypes and femaleness of self–pollinated progeny families from a monoecious selection in an S2BC2F1 population produced by crosses between 03–009 and 03–336. Black boxes represent homozygous 03–336 segments, gray boxes indicate heterozygous 03–009/03–336 regions, and white boxes represent homozygous 03–009 segments. Marker positions and names are indicated above the boxes. Indices of femaleness (expressed as the percentage of female flowers per plant) are shown to the right of the boxes. (PDF) [file pone.0296675.s006.pdf]

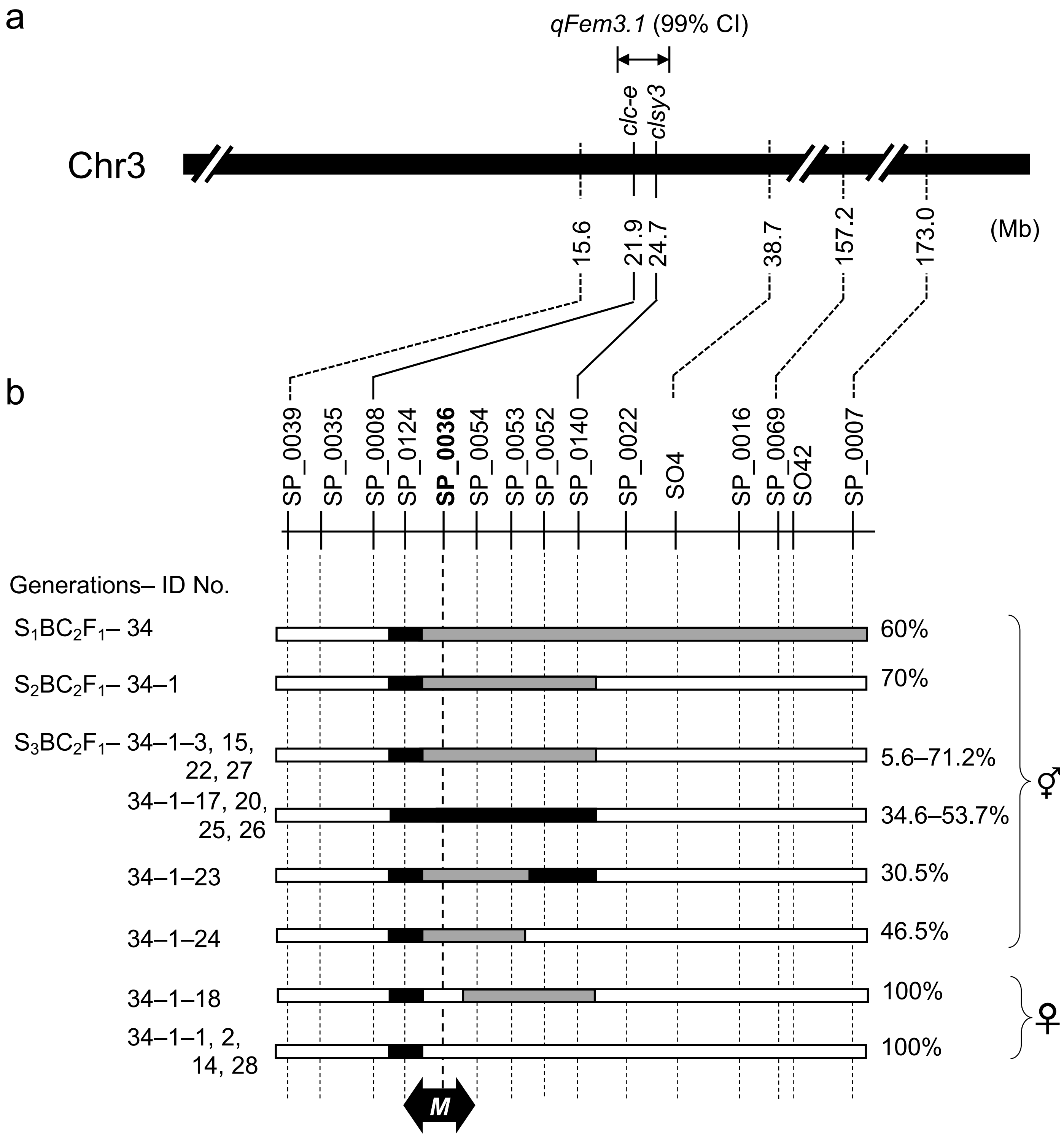

S6 Fig. Schematic diagrams showing the physical position of a chromosomal region harboring the *qFem3.1 and M* loci. a. A schematic diagram showing the physical positions of *qFem3.1* and DNA markers on a pseudomolecule, Chr3. **b.** Graphical genotypes and femaleness of self–pollinated progeny families from a monoecious selection in an $S_2BC_2F_1$ population produced by crosses between 03–009 and 03–336. Black boxes represent homozygous 03–336 segments, gray boxes indicate heterozygous 03–009/03–336 regions, and white boxes represent homozygous 03–009 segments. Marker positions and names are indicated above the boxes. Indices of femaleness (expressed as the percentage of female flowers per plant) are shown to the right of the boxes.
