# Supplementary material for: QTL analysis of femaleness in monoecious spinach and fine mapping of a major QTL using an updated version of chromosome-scale pseudomolecules
Source: PLoS One. 2024 Feb 23;19(2):e0296675. doi: 10.1371/journal.pone.0296675 (PMC10890751; doi:10.1371/journal.pone.0296675)
Supplement: S7 Fig — Black boxes represent homozygous 03–336 segments, gray boxes indicate heterozygous 03–009/03–336 regions, and white boxes represent homozygous 03–009 segments. Markers are indicated above the boxes. Indices of femaleness (expressed as the percentage of female flowers per plant) are shown to the right of the boxes. (PDF) [file pone.0296675.s007.pdf]

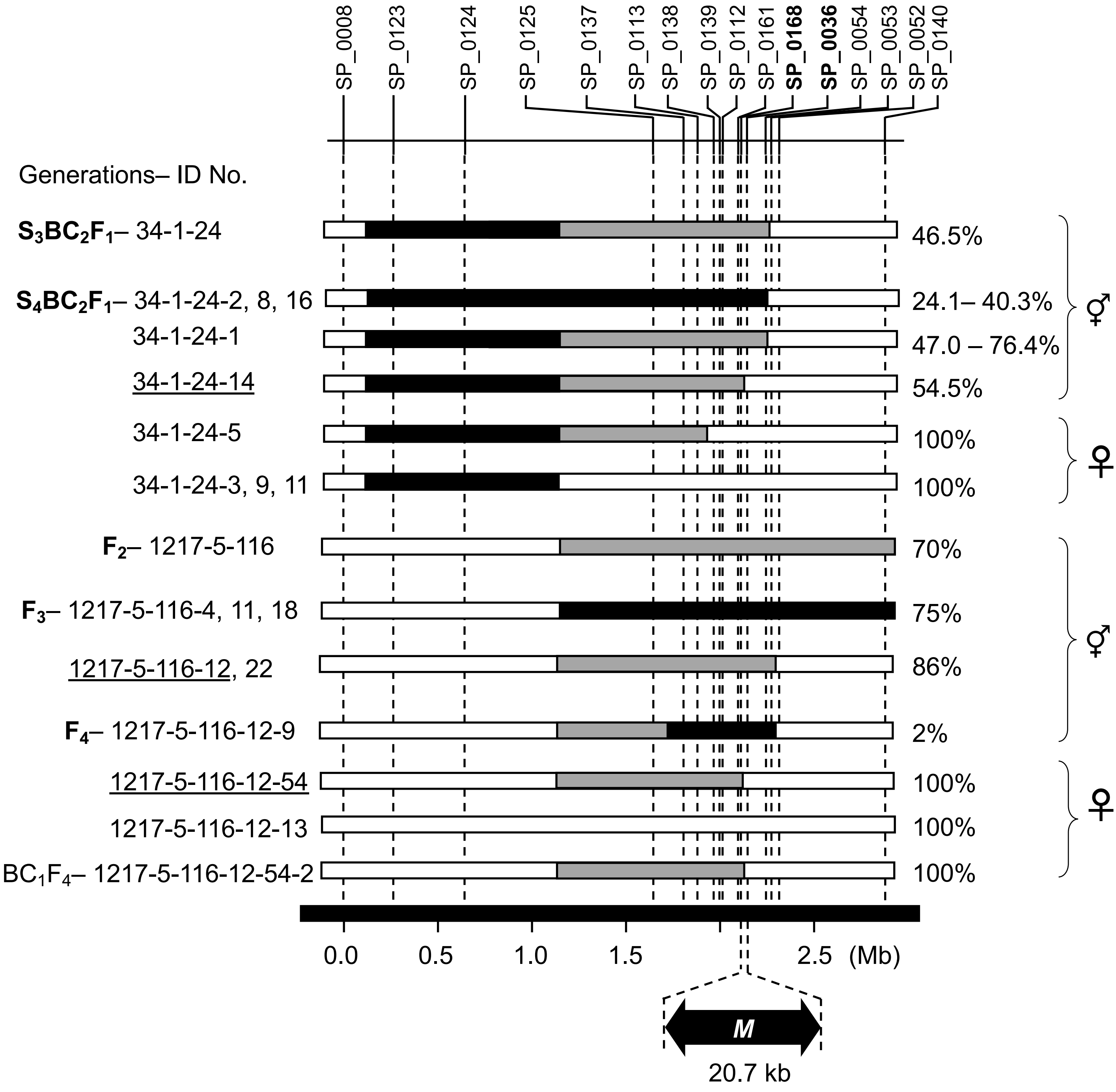

S7 Fig. Graphical genotypes and femaleness of progeny families from monoecious selections in a $S_3BC_2F_1$ and a F_2 population produced by crosses between 03–009 and 03–336. Black boxes represent homozygous 03–336 segments, gray boxes indicate heterozygous 03–009/03–336 regions, and white boxes represent homozygous 03–009 segments. Markers are indicated above the boxes. Indices of femaleness (expressed as the percentage of female flowers per plant) are shown to the right of the boxes.
