# Supplementary material for: QTL analysis of femaleness in monoecious spinach and fine mapping of a major QTL using an updated version of chromosome-scale pseudomolecules
Source: PLoS One. 2024 Feb 23;19(2):e0296675. doi: 10.1371/journal.pone.0296675 (PMC10890751; doi:10.1371/journal.pone.0296675)
Supplement: S8 Fig — (PDF) [file pone.0296675.s008.pdf]

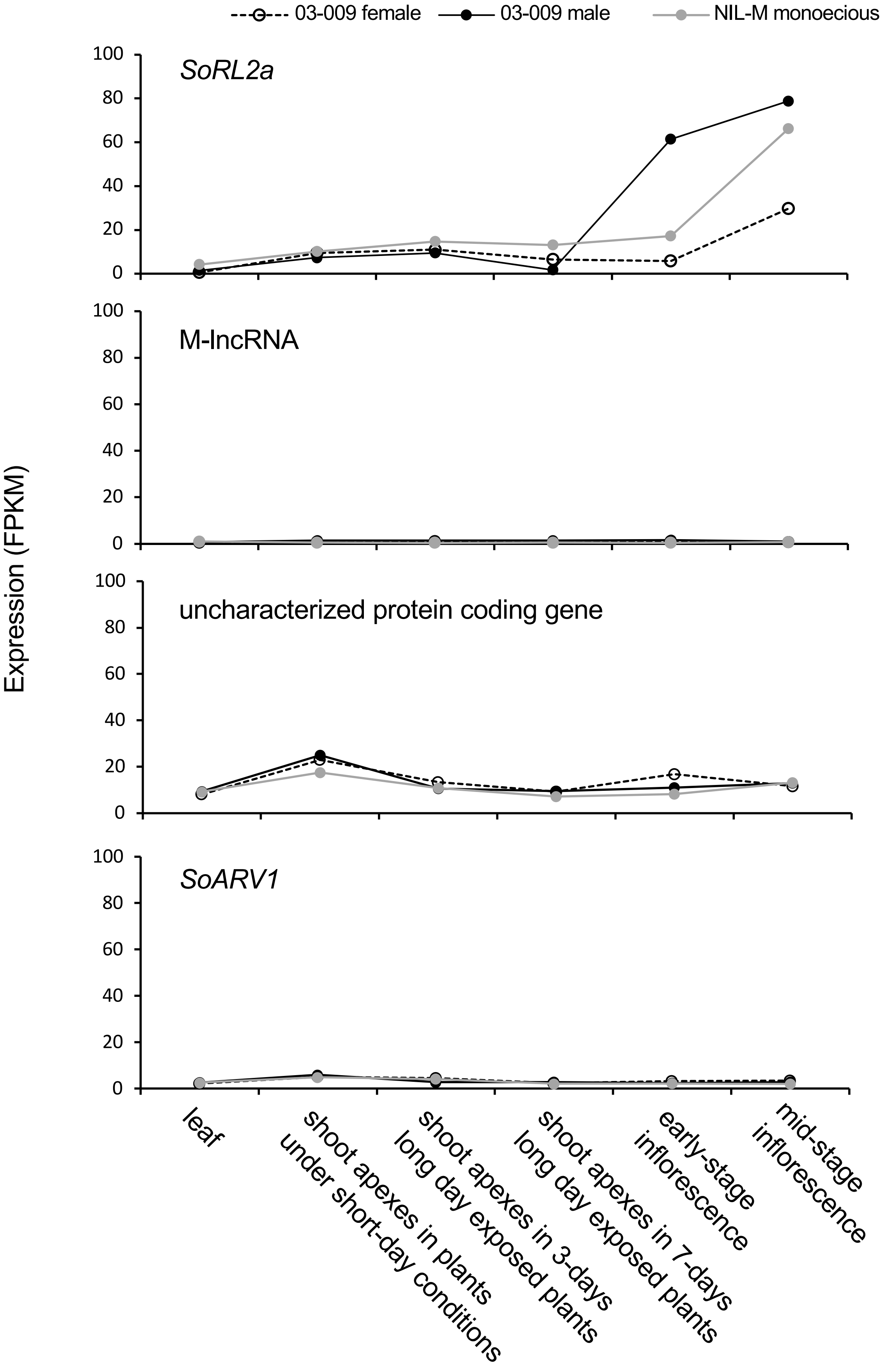

S8 Fig. Expression levels of the genes located in the 19.5-kb chromosomal region candidate for the monoecious locus measured by RNA-seq.
