# Supplementary material for: QTL analysis of femaleness in monoecious spinach and fine mapping of a major QTL using an updated version of chromosome-scale pseudomolecules
Source: PLoS One. 2024 Feb 23;19(2):e0296675. doi: 10.1371/journal.pone.0296675 (PMC10890751; doi:10.1371/journal.pone.0296675)
Supplement: S9 Fig — S. oleracea (So), Beta vulgaris (Bv), Arabidopsis thaliana (At), Diospyros kaki (Dk), Antirrhinum majus (Am) and Oryza sativa (Os). Bootstrap values above 50% from 1,000 iterations are indicated below the nodes. (PDF) [file pone.0296675.s009.pdf]

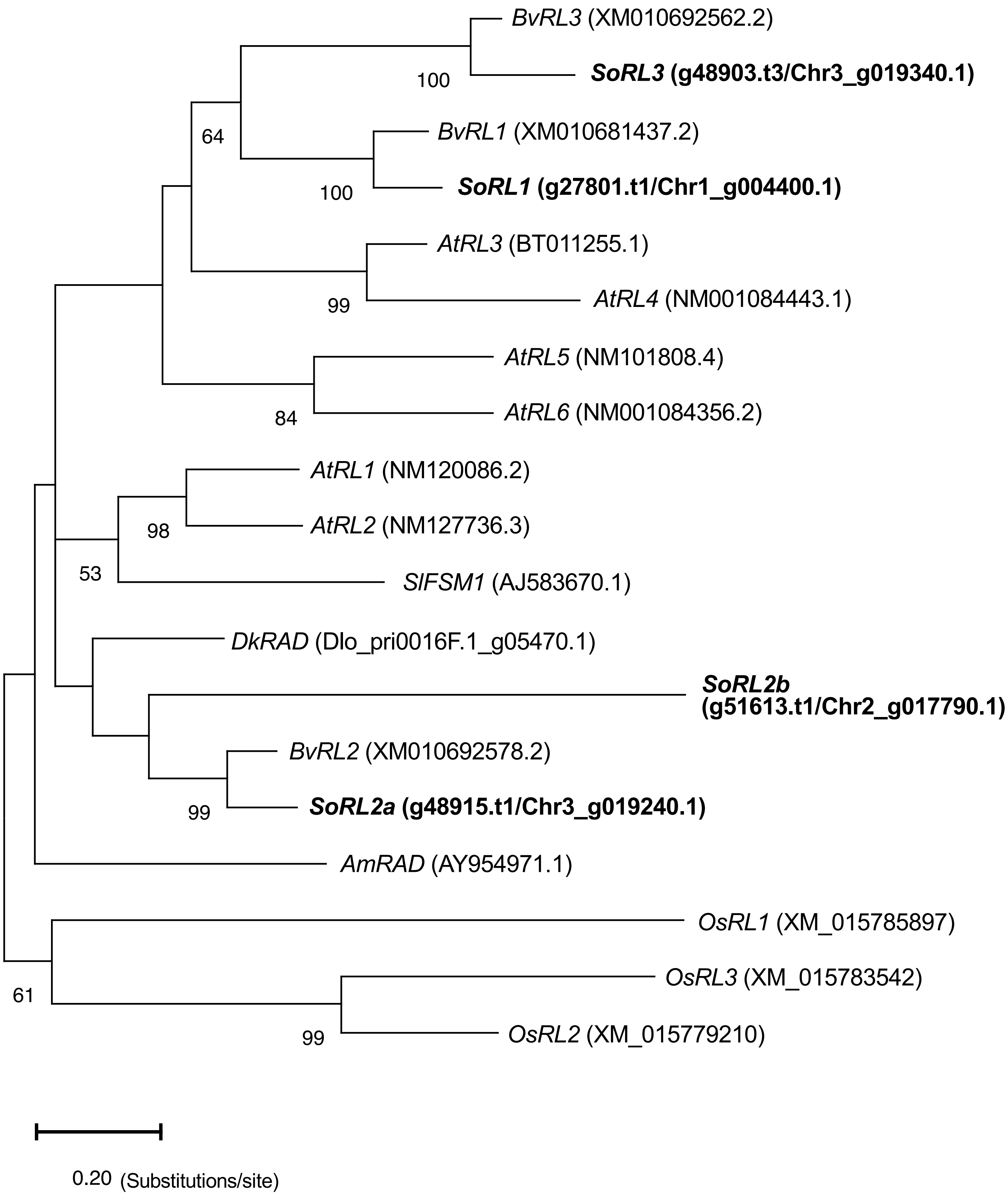

S9 Fig. Maximum likelihood tree of RADIALS-like genes. *S. oleracea* (*So*), *Beta vulgaris* (*Bv*), *Arabidopsis thaliana* (*At*), *Diospyros kaki* (*Dk*), *Antirrhinum majus* (*Am*) and *Oryza sativa* (*Os*). Bootstrap values above 50% from 1,000 iterations are indicated below the nodes.
