# Supplementary material for: QTL analysis of femaleness in monoecious spinach and fine mapping of a major QTL using an updated version of chromosome-scale pseudomolecules
Source: PLoS One. 2024 Feb 23;19(2):e0296675. doi: 10.1371/journal.pone.0296675 (PMC10890751; doi:10.1371/journal.pone.0296675)
Supplement: S10 Fig — a-c, Hematoxylin-eosin-stained sections of flower(s) from a male (a and b) and a female (c) plant. Two male flowers at different developmental stages are shown in panel a and numbered 1 and 2; a’-c’, In situ hybridization of flower sections from a male (a’ and b’) and a female (c’) plant with anti-sense SoRL2a riboprobes; a”-c”, Flower sections from a male (a") and a female (b" and c") plant probed with sense SoRL2a. E, epidermis; En, endothecium; ML, middle layer; MMC, microspore mother cells; T, tapetum; Tds, tetrads; G, gynoecium; II, inner integument; OI, outer integument; Nu, nucellus; Ch, chalaza. (PDF) [file pone.0296675.s010.pdf]

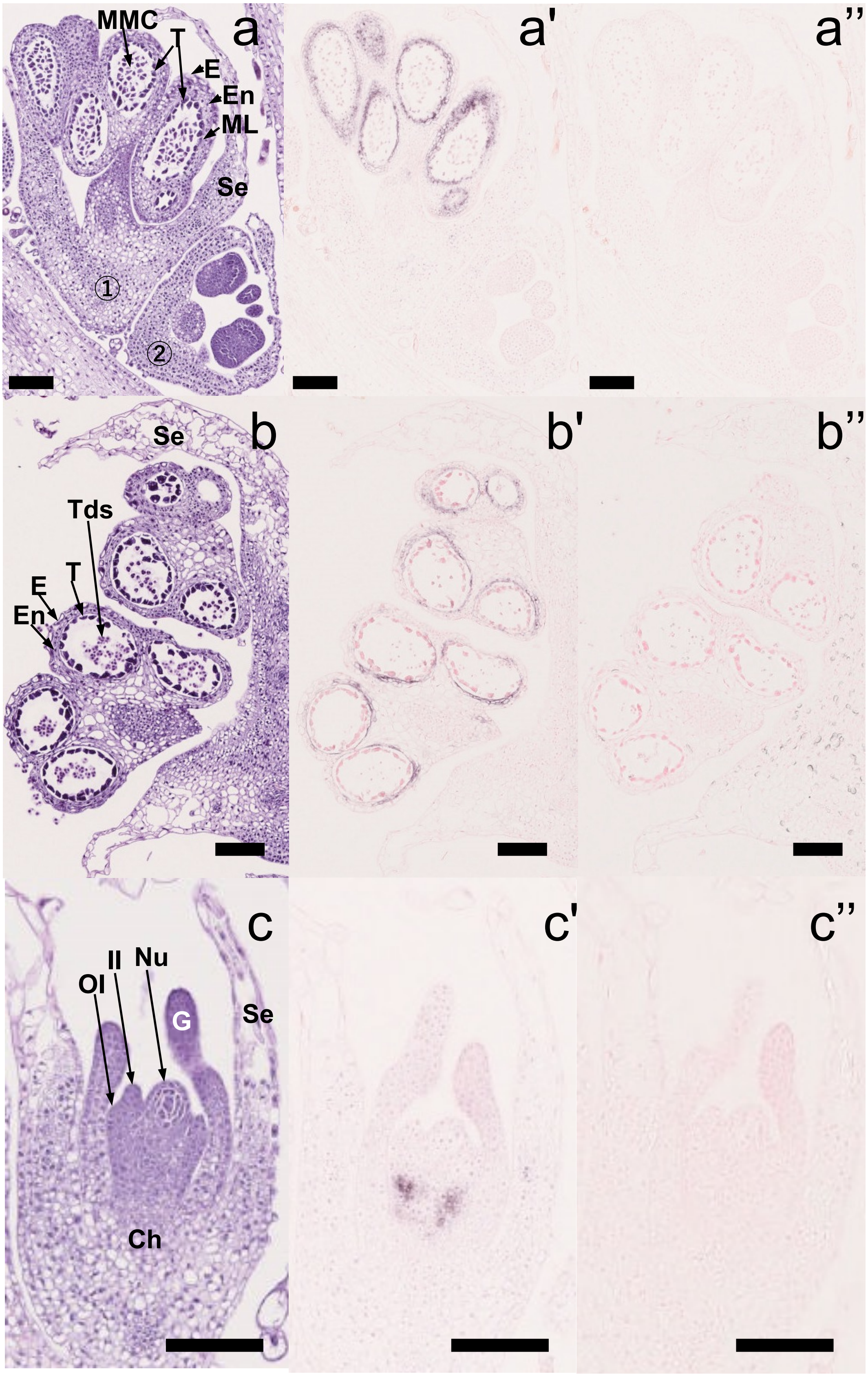

S10 Fig. Spatial expression analysis of *SoRL2a*. a-c, Hematoxylin-eosin-stained sections of flower(s) from a male (**a** and **b**) and a female (**c**) plant. Two male flowers at different developmental stages are shown in panel **a** and numbered 1 and 2; **a'-c'**, *In situ* hybridization of flower sections from a male (**a'** and **b'**) and a female (**c'**) plant with anti-sense *SoRL2a* riboprobes; **a''-c''**, Flower sections from a male (**a''**) and a female (**b''** and **c''**) plant probed with sense *SoRL2a*. E, epidermis; En, endothecium; ML, middle layer; MMC, microspore mother cells; T, tapetum; Tds, tetrads; G, gynoecium; **II**, inner integument; OI, outer integument; Nu, nucellus; Ch, chalaza. Note: Above, in S6 Fig, you use capital letters for the panels (i.e., **A** and **B**). Please standardize the usage throughout the Supporting Information.
