# Supplementary material for: QTL analysis of femaleness in monoecious spinach and fine mapping of a major QTL using an updated version of chromosome-scale pseudomolecules
Source: PLoS One. 2024 Feb 23;19(2):e0296675. doi: 10.1371/journal.pone.0296675 (PMC10890751; doi:10.1371/journal.pone.0296675)
Supplement: S11 Fig — Arrowheads and horizontal bars indicate average numbers and ranges, respectively, of femaleness of the parental lines, 03–009 and 03–336, the F1 progeny, and NIL-M (S2BC5F1). (PDF) [file pone.0296675.s011.pdf]

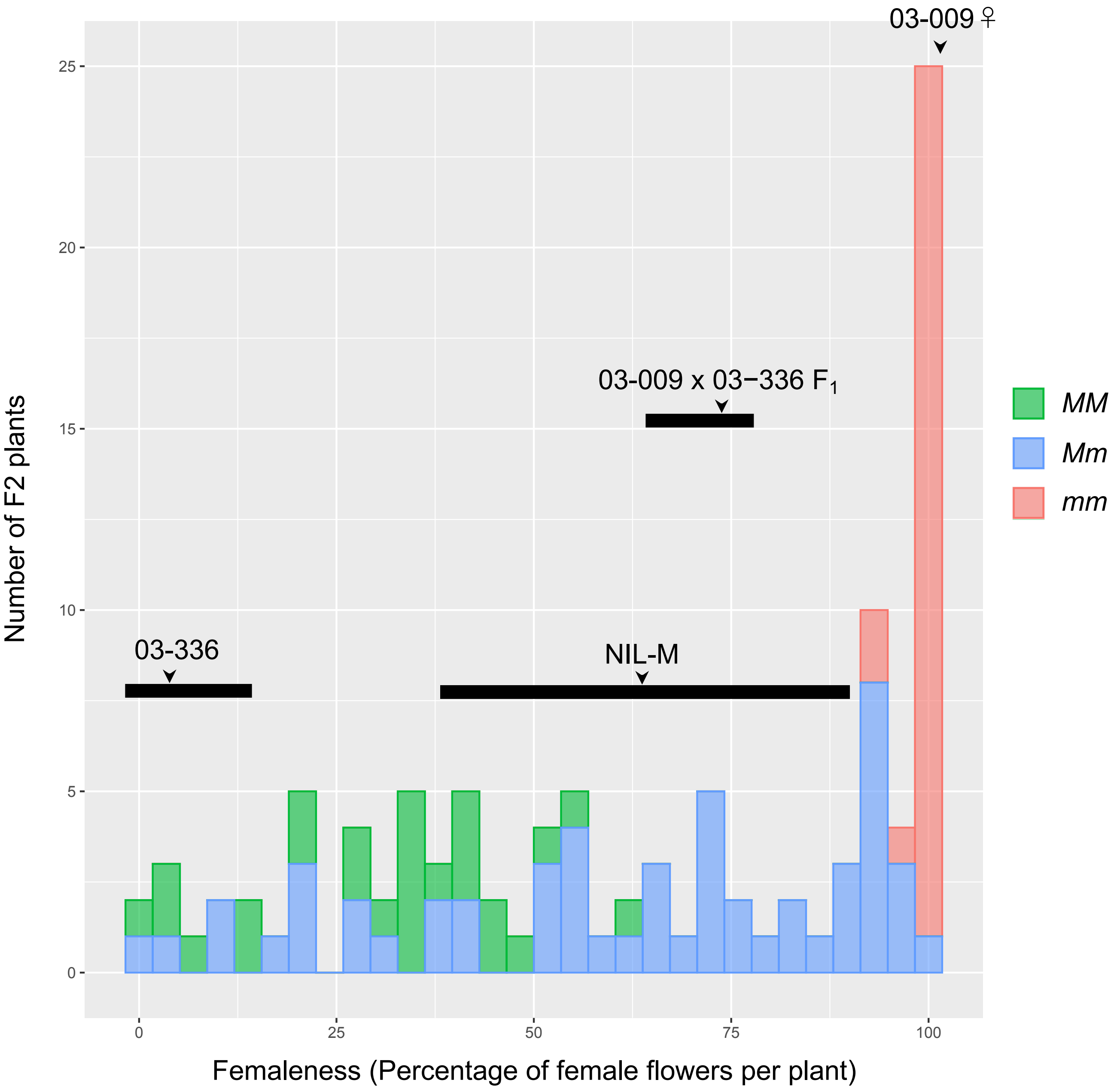

S11 Fig. Frequency distribution of the femaleness in the 96 F_2 progeny plants from the cross between 03-009 and 03-336. Arrowheads and horizontal bars indicate average numbers and ranges, respectively, of femaleness of the parental lines, 03-009 and 03-336, the F_1 progeny, and NIL-M ($S_2BC_5F_1$).
