# Supplementary material for: QTL analysis of femaleness in monoecious spinach and fine mapping of a major QTL using an updated version of chromosome-scale pseudomolecules
Source: PLoS One. 2024 Feb 23;19(2):e0296675. doi: 10.1371/journal.pone.0296675 (PMC10890751; doi:10.1371/journal.pone.0296675)
Supplement: S12 Fig — A, homozygous for the 03-009-derived allele; H, heterozygous for the 03–009 and 03-336-derived alleles; B, homozygous for the 03-336-derived allele. (PDF) [file pone.0296675.s012.pdf]

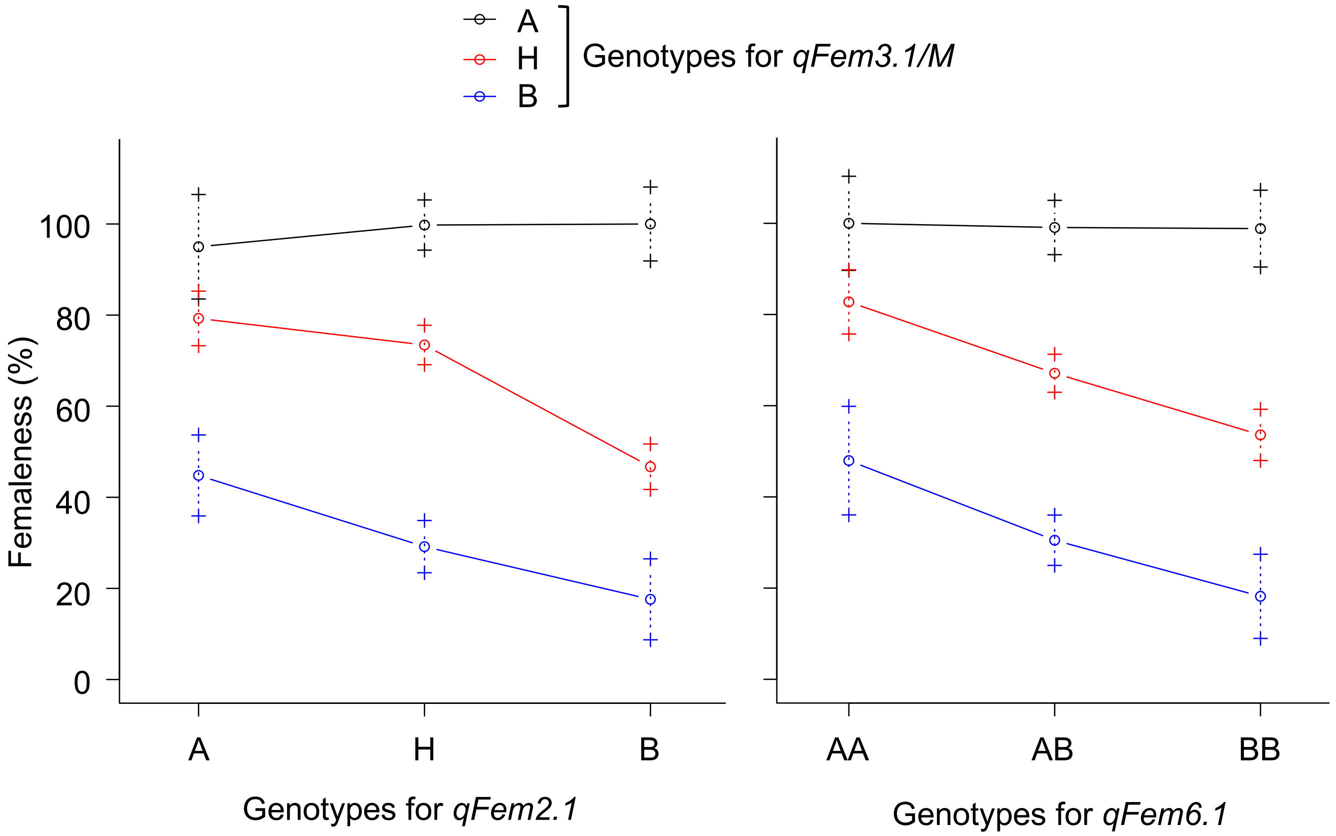

S12 Fig. Means of femaleness scores in the 03-009 x 03-336 F_2 population against genotypes at the QTLs. A, homozygous for the 03-009-derived allele; H, heterozygous for the 03-009 and 03-336-derived alleles; B, homozygous for the 03-336-derived allele.
