# Supplementary material for: QTL analysis of femaleness in monoecious spinach and fine mapping of a major QTL using an updated version of chromosome-scale pseudomolecules
Source: PLoS One. 2024 Feb 23;19(2):e0296675. doi: 10.1371/journal.pone.0296675 (PMC10890751; doi:10.1371/journal.pone.0296675)
Supplement: S13 Fig — 03–336, highly male monoecious; NIL-M, highly female monoecious; 03–009, female. (PDF) [file pone.0296675.s013.pdf]

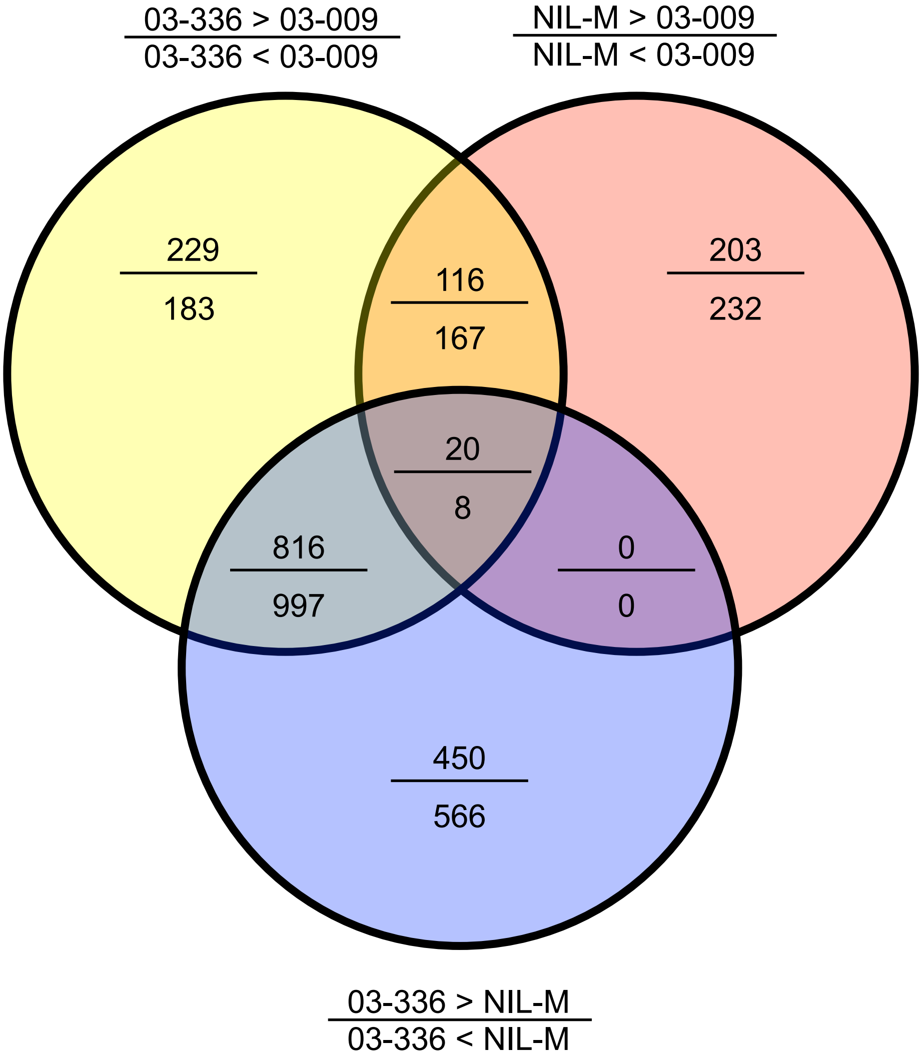

S13 Fig. Venn diagrams showing the genes differentially expressed between early-stage inflorescences from the spinach lines 03-336, NIL-M and 03-009. 03-336, highly male monoecious; NIL-M, highly female monoecious; 03-009, female.
