# Supplementary material for: QTL analysis of femaleness in monoecious spinach and fine mapping of a major QTL using an updated version of chromosome-scale pseudomolecules
Source: PLoS One. 2024 Feb 23;19(2):e0296675. doi: 10.1371/journal.pone.0296675 (PMC10890751; doi:10.1371/journal.pone.0296675)
Supplement: S1 Table — (PDF) [file pone.0296675.s014.pdf]

S1 Table. Status of genome assemblies for spinach line 03-009 in the five assembly steps.

Assembly ID		Spol_r0.0	Spol_r0.1	Spol_r0.2	Spol_r1.0	SOL_r2.0_pseudomolecule (Present study)	SOL_r1.0_pseudomolecule (Hirakawa et al. 2021)	Monoe-Viroflay, Chrs1-6 (Cai et al. 2021)	Viroflay XX, Chrs1-6 (GWHBHEW00000000) (Ma et al. 2022)	Cornell-No.9 YY, Chrs1-6 (GWHBGBP00000000) (Ma et al. 2022)
Softwares used for polishing and scaffoldings		Arrow	Arrow/Racon	Arrow/Racon/Pilon	Arrow/Racon/Pilon/SALSA2.3	-	-	-	-	-
		primary contigs	FALCON-Unzip-Arrow-Racon (primary)	FALCON-Unzip-Arrow-Rcon-Pilon1 (primary)	FALCON-Unzip-Arrow-Racon-Pilon1_SALSA2.3 (primary) (GATC,GANTC)	-	-	-	-	-
Total	Number of sequences	508	508	508	323	6	6	6	6	6
	Total length (bases)	934,335,220	935,826,662	935,243,733	935,351,733	879,181,464	687,989,365	879,228,028	894,318,217	950,323,615
	Average length (bases)	1,839,243	1,842,178	1,841,031	2,895,826	146,530,244	114,664,894	146,538,005	149,053,036	158,387,269
	Max length (bases)	25,783,435	25,813,488	25,798,206	151,927,733	188,023,793	146,198,663	189,052,256	192,529,778	217,806,951
	Min length (bases)	20,316	20,115	20,064	20,064	115,602,668	85,406,467	114,788,624	122,088,003	126,542,630
	N50 length (bases)	6,737,553	6,748,817	6,744,358	74,575,922	152,476,739	124,699,619	151,450,279	151,123,541	161,159,065
	A	290,447,427	291,039,965	290,803,409	290,941,685	273,324,180	213,871,762	273,595,536	277,782,862	294,688,969
	T	290,412,320	291,054,086	290,792,703	290,654,427	273,420,130	213,683,023	273,504,425	278,177,621	294,552,701
	G	176,743,545	176,875,678	176,830,653	176,850,448	165,753,833	129,307,420	166,010,789	169,240,967	178,282,743
	C	176,731,928	176,856,933	176,816,968	176,797,173	165,682,821	129,404,416	166,110,978	168,983,467	178,321,102
	n	0	0	0	0	0	0	0	0	0
	N	0	0	0	108,000	1,000,500	1,722,744	6,300	133,300	4,478,100
	others	0	0	0	0	0	0	0	0	0
	Total	934,335,220	935,826,662	935,243,733	935,351,733	879,181,464	687,989,365	879,228,028	894,318,217	950,323,615
	GC%	37.8	37.8	37.8	37.8	37.7	37.6	37.8	37.8	37.5
	Total (ATGC)	934,335,220	935,826,662	935,243,733	935,243,733	878,180,964	686,266,621	879,221,728	894,184,917	945,845,515
	GC% (ATGC)	37.8	37.8	37.8	37.8	37.7	37.7	37.8	37.8	37.7
≥100 kb	Number of sequences	351	351	351	173	6	6	6	6	6
	Total length (bases)	925,797,883	927,233,062	926,656,582	927,358,881	879,181,464	687,989,365	879,228,028	894,318,217	950,323,615
	Average length (bases)	2,637,601	2,641,690	2,640,047	5,360,456	146,530,244	114,664,894	146,538,005	149,053,036	158,387,269
≥500 kb	Number of sequences	215	215	215	71	6	6	6	6	6
	Total length (bases)	895,499,978	896,842,377	896,285,506	905,737,654	879,181,464	687,989,365	879,228,028	894,318,217	950,323,615
	Average length (bases)	4,165,116	4,171,360	4,168,770	12,756,868	146,530,244	114,664,894	146,538,005	149,053,036	158,387,269
≥1 Mb	Number of sequences	176	176	176	55	6	6	6	6	6
	Total length (bases)	867,424,952	868,702,283	868,164,482	895,568,072	879,181,464	687,989,365	879,228,028	894,318,217	950,323,615
	Average length (bases)	4,928,551	4,935,808	4,932,753	16,283,056	146,530,244	114,664,894	146,538,005	149,053,036	158,387,269
≥2 Mb	Number of sequences	127	127	127	52	6	6	6	6	6
	Total length (bases)	793,092,607	794,256,011	793,763,293	891,068,213	879,181,464	687,989,365	879,228,028	894,318,217	950,323,615
	Average length (bases)	6,244,824	6,253,984	6,250,105	17,135,927	146,530,244	114,664,894	146,538,005	149,053,036	158,387,269
≥3 Mb	Number of sequences	94	94	94	36	6	6	6	6	6
	Total length (bases)	715,136,591	716,176,580	715,730,229	850,323,222	879,181,464	687,989,365	879,228,028	894,318,217	950,323,615
	Average length (bases)	7,607,836	7,618,900	7,614,151	23,620,090	146,530,244	114,664,894	146,538,005	149,053,036	158,387,269
≥4 Mb	Number of sequences	73	73	73	28	6	6	6	6	6
	Total length (bases)	644,738,333	645,651,536	645,248,931	822,532,938	879,181,464	687,989,365	879,228,028	894,318,217	950,323,615
	Average length (bases)	8,832,032	8,844,542	8,839,026	29,376,176	146,530,244	114,664,894	146,538,005	149,053,036	158,387,269
≥5 Mb	Number of sequences	61	61	61	25	6	6	6	6	6
	Total length (bases)	591,348,863	592,179,204	591,808,942	809,195,960	879,181,464	687,989,365	879,228,028	894,318,217	950,323,615
	Average length (bases)	9,694,244	9,707,856	9,701,786	32,367,838	146,530,244	114,664,894	146,538,005	149,053,036	158,387,269
≥10 Mb	Number of sequences	22	22	22	15	6	6	6	6	6
	Total length (bases)	330,902,046	331,350,134	331,139,376	728,652,758	879,181,464	687,989,365	879,228,028	894,318,217	950,323,615
	Average length (bases)	15,041,002	15,061,370	15,051,790	48,576,851	146,530,244	114,664,894	146,538,005	149,053,036	158,387,269
≥50 Mb	Number of sequences	0	0	0	7	6	6	6	6	6
	Total length (bases)	0	0	0	592,667,731	879,181,464	687,989,365	879,228,028	894,318,217	950,323,615
	Average length (bases)	0	0	0	84,666,819	146,530,244	114,664,894	146,538,005	149,053,036	158,387,269
≥100 Mb	Number of sequences	0	0	0	2	6	4	6	6	6
	Total length (bases)	0	0	0	261,120,827	879,181,464	514,336,635	879,228,028	894,318,217	950,323,615
	Average length (bases)	0	0	0	130,560,414	146,530,244	128,584,159	146,538,005	149,053,036	158,387,269
BUSCO v5.3.2 (embryophyta_odb10; 1614)	Complete%	97.1	96.9	97.5	97.4	97.0	81.5	97.8	97.8	94.9
	Complete and single-copy%	93.3	93.1	93.5	93.6	94.4	79.7	96.0	96.0	92.1
	Complete and duplicated%	3.8	3.8	4.0	3.8	2.6	1.8	1.8	1.8	2.8
	Fragmented%	1.1	1.7	1.2	1.3	1.3	1.7	0.8	0.7	2.6
	Missing%	1.8	1.4	1.3	1.3	1.7	16.8	1.4	1.5	2.5
LAI (LTR Assembly Index)		-	-	-	-	19.94	17.08	20.42	19.58	-
