# Supplementary material for: QTL analysis of femaleness in monoecious spinach and fine mapping of a major QTL using an updated version of chromosome-scale pseudomolecules
Source: PLoS One. 2024 Feb 23;19(2):e0296675. doi: 10.1371/journal.pone.0296675 (PMC10890751; doi:10.1371/journal.pone.0296675)
Supplement: S2 Table — (PDF) [file pone.0296675.s015.pdf]

S2 Table. Hi-C mate-pair reads used in this study.

Accession number	Platform	Library	Read length (nt)	Sample	Total number of reads	Total bases (nt)
DRX477737	HiSeq X	Paired-end (read1)	150	03-009	305,393,215	45,808,982,250
DRX477737	HiSeq X	Paired-end (read2)	150	03-009	305,393,215	45,808,982,250

All data is available under DRA016730 (BioProject: PRJDB10697).
