# Supplementary material for: QTL analysis of femaleness in monoecious spinach and fine mapping of a major QTL using an updated version of chromosome-scale pseudomolecules
Source: PLoS One. 2024 Feb 23;19(2):e0296675. doi: 10.1371/journal.pone.0296675 (PMC10890751; doi:10.1371/journal.pone.0296675)
Supplement: S3 Table — (PDF) [file pone.0296675.s016.pdf]

S3 Table. Summary of whole genome sequencing reads used for variant discovery.

Accession number	Platform	Library	Read length (bp)	Sample	Total number of reads	Total bases
DRX401201	HiSeq 2500	Paired-end (read1)	251	03-009	51,716,825	12,980,923,075
DRX401201	HiSeq 2500	Paired-end (read2)	251	03-009	51,716,825	12,980,923,075
DRX401202	HiSeq 2500	Paired-end (read1)	251	03-336	50,911,361	12,778,751,611
DRX401202	HiSeq 2500	Paired-end (read2)	251	03-336	50,911,361	12,778,751,611

All data is available under DRA015088 (BioProject: PRJDB14728).
