# Supplementary material for: QTL analysis of femaleness in monoecious spinach and fine mapping of a major QTL using an updated version of chromosome-scale pseudomolecules
Source: PLoS One. 2024 Feb 23;19(2):e0296675. doi: 10.1371/journal.pone.0296675 (PMC10890751; doi:10.1371/journal.pone.0296675)
Supplement: S4 Table — (PDF) [file pone.0296675.s017.pdf]

S4 Table. Primer sequences and annealing temperatures of the spinach genetic markers used for fine mapping of the M-locus.

marker ID	marker type (enzyme)	dominant / codominant	length of variant fragments (bp) 03-009/03-336	primer sequence (5'-3')	annealing temperature
SP_0036	dCAPS (DdeI)	codominant	222+29/251	AGCATTTGTGTAAGATCGCC	53
				GTTGTCATCTAATGAAACCGATTAGCTA	
SP_0052	CAPS(Rsal)	codominant	593/310+284	ATCGACCCATTAAGGCTGATC	54
				GGTTATGGCTGCTGTTGCTATG	
SP_0053	SCAR	dominant	null/1066	TCCTGCTTGAGCTTATAGAGC	55
				GGCTCTTTGAATCTCCAATG	
SP_0054	SCAR	dominant	null/730	ACCATGGATTAGAACTAGAGCC	55
				CGTAAGTAGAGGATACCCAAGTG	
SP_0112	dCAPS (HapII)	codominant	220/241	TCCTTGAAGGTGGTGTCTCTG	56
				AAGGGACTGCAAACTGTCACC	
SP_0113	dCAPS (RsaI)	codominant	152/125+27	TAGATTCATCACTGTTTGAGCCAACGTA	58
				CACAGAATTGCAACGAGAAGACG	
SP_0123	dCAPS (AluI)	codominant	143/167	TTGGCCATTTTGTACCGGGTTTAGC	55
				GGATGAGGATTAGCTCCAATTCTCTGAG	
SP_0124	CAPS (TaqI)	codominant	123+312/435	CGTAAAGTCAAGTGAGTAAGATTGCC	51
				GAGATCGGTATAGGATCTTGCTAC	
SP_0125	dCAPS (HinfI)	codominant	285/258+27	GAGGGACTGGATAGTACAGAGACAATCG	57
				CTCTTCCTCTCTCTCCTCAACACTGAC	
SP_0137g	allele-specific	dominant	255/–	CACTGCCATTTTCCTTTTTCATG	52
				AAGCACGTATGTCGAGCAAC	
SP_0137c	allele-specific	dominant	–/255	CACTGCCATTTTCCTTTTTCATC	52
				AAGCACGTATGTCGAGCAAC	
SP_0138g	allele-specific	dominant	198/–	CGAACATAACCATAGAAGAACGG	50
				TAATCTCAACGTTGCCCATG	
SP_0138c	allele-specific	dominant	–/198	CGAACATAACCATAGAAGAACGC	50
				TAATCTCAACGTTGCCCATG	
SP_0139c	allele-specific	dominant	297/–	CATTGACAAGCGTGTCCATTCAAAC	55
				ACGCCTGATTCAAATCTCGGC	
SP_0139t	allele-specific	dominant	–/297	CATTGACAAGCGTGTCCATTCAAAC	55
				ACGCCTGATTCAAATCTCGGT	
SP_0161c	allele-specific	dominant	271/–	ACTAACAGCAACATCCTCACTAC	54
				CGTCGTGAAGGTGGATGC	
SP_0161t	allele-specific	dominant	–/271	ACTAACAGCAACATCCTCACTAC	54
				CGTCGTGAAGGTGGATGT	
SP_0168	SCAR	dominant	–/193	GGTTGTTTTTATCCACAAGCTTGTT	54
				CATTACTTCCAAGGTTGAAACTATCG	
SP_0169	dCAPS (DdeI)	codominant	239/270	GGTGTCGACATGTTAGTCCG	54
				GAAACATTTACCACTTCTTTGGATACCC	
SP_0180	SCAR	codominant	325/377	TGGATTCCTTCAGTTTCAGCAG	52
				TTCGGAGGGAGTATAAGCAT	
SP_0181	SCAR	codominant	228/202	GATGACAACAAAATGGTATAACCG	53
				TGCAAACGCCTACTTCCAGTAC	
