# Supplementary material for: QTL analysis of femaleness in monoecious spinach and fine mapping of a major QTL using an updated version of chromosome-scale pseudomolecules
Source: PLoS One. 2024 Feb 23;19(2):e0296675. doi: 10.1371/journal.pone.0296675 (PMC10890751; doi:10.1371/journal.pone.0296675)
Supplement: S5 Table — (PDF) [file pone.0296675.s018.pdf]

S5 Table. Primer sequences and annealing temperatures of the spinach genetic markers flanking the QTLs controlling monoecious expression.

marker ID	chromosomal location	target locus	marker type (enzyme)	length of variant fragments (bp) 03-009 / 03-336	primer sequence (5'-3')	annealing temperatur
SP_0205	Chr3: 24,035,704-24,035,966	*qFem3.1/M*	SCAR	308/198	TTGGTGTGCGAAATACCCTG<br>CACAGATAGCGTGTATGTAAGACTAG	53
SP_0319	Chr2: 572,605-572,774	*qFem2.1*	SCAR	208/235	TCTGGTGAGGGGAAAGTTGC<br>CTCGAGCCCCAAAAATCAAG	53
SP_0325	Chr6: 80,439,150-80,439,285	*qFem6.1*	dCAPS (EcoRV)	110/136	GTTATATGCTTAGTGTTATATTTTCTCTGTGG<br>ACTTTCTTTTTAACAGTCACGGATTTGAT	62
