# Supplementary material for: QTL analysis of femaleness in monoecious spinach and fine mapping of a major QTL using an updated version of chromosome-scale pseudomolecules
Source: PLoS One. 2024 Feb 23;19(2):e0296675. doi: 10.1371/journal.pone.0296675 (PMC10890751; doi:10.1371/journal.pone.0296675)
Supplement: S6 Table — (PDF) [file pone.0296675.s019.pdf]

S6 Table. Primers used for the RT-qPCR assay.

Target_gene	primer sequence (5'-3')	annealing temperature
Actin-7	CCTCTGTTTCTTCAATTAGCAGGGC	55
	GCAAACCCAGCCTTAACCATCC	
SoRL2a	GCCAAGTGCCCTTTCCTAACTA	53
	GATCTATCTTCAACAACAGCGCG	
