# Supplementary material for: QTL analysis of femaleness in monoecious spinach and fine mapping of a major QTL using an updated version of chromosome-scale pseudomolecules
Source: PLoS One. 2024 Feb 23;19(2):e0296675. doi: 10.1371/journal.pone.0296675 (PMC10890751; doi:10.1371/journal.pone.0296675)
Supplement: S7 Table — (PDF) [file pone.0296675.s020.pdf]

S7 Table. RNA-Seq reads used for transcriptome analysis.

Bioproject	Accession number	Platform	Library	Read length (bp)	Total number of reads	Total bases (nt)
PRJDB6306	DRX094096	HiSeq 2500	Paired-end	100	76,758,820	7,675,882,000
PRJDB6306	DRX094097	HiSeq 2500	Paired-end	100	220,107,592	22,010,759,200
PRJDB6306	DRX094098	HiSeq 2500	Paired-end	100	55,845,988	5,584,598,800
PRJDB6306	DRX094099	HiSeq 2500	Paired-end	100	72,440,368	7,244,036,800
PRJDB6306	DRX094100	HiSeq 2500	Paired-end	100	85,455,984	8,545,598,400
PRJDB6306	DRX094101	HiSeq 2500	Paired-end	100	72,082,064	7,208,206,400
PRJDB6306	DRX094102	HiSeq 2500	Paired-end	100	166,204,340	16,620,434,000
PRJDB6306	DRX094103	HiSeq 2500	Paired-end	100	103,718,646	10,371,864,600
PRJDB6306	DRX094104	HiSeq 2500	Paired-end	100	233,760,976	23,376,097,600
PRJDB6306	DRX094105	HiSeq 2500	Paired-end	100	62,593,332	6,259,333,200
PRJDB6306	DRX094106	HiSeq 2500	Paired-end	100	88,093,298	8,809,329,800
PRJDB6306	DRX094107	HiSeq 2500	Paired-end	100	46,359,908	4,635,990,800
PRJDB6306	DRX094108	HiSeq 2500	Paired-end	100	74,917,390	7,491,739,000
PRJDB6306	DRX094109	HiSeq 2500	Paired-end	100	108,186,454	10,818,645,400
PRJDB6306	DRX094110	HiSeq 2500	Paired-end	100	90,067,040	9,006,704,000
PRJDB6306	DRX094111	HiSeq 2500	Paired-end	100	176,752,478	17,675,247,800
PRJDB6306	DRX094112	HiSeq 2500	Paired-end	100	162,767,124	16,276,712,400
PRJDB6306	DRX094113	HiSeq 2500	Paired-end	100	50,609,258	5,060,925,800
PRJDB10697	DRX477738	NextSeq 1000	Single-end	100	18,841,704	1,867,638,799
PRJDB10697	DRX477739	NextSeq 1000	Single-end	100	20,409,574	2,021,507,559
PRJDB10697	DRX477740	NextSeq 1000	Single-end	100	21,520,989	2,132,800,235
PRJDB10697	DRX477741	NextSeq 1000	Single-end	100	19,256,515	1,905,293,261
PRJDB10697	DRX477742	NextSeq 1000	Single-end	100	19,834,766	1,963,116,363
PRJDB10697	DRX477743	NextSeq 1000	Single-end	100	20,259,163	2,007,167,135
PRJDB10697	DRX477744	NextSeq 1000	Single-end	100	18,094,460	1,797,599,833
PRJDB10697	DRX477745	NextSeq 1000	Single-end	100	21,108,293	2,096,967,616
PRJDB10697	DRX477746	NextSeq 1000	Single-end	100	18,340,528	1,822,064,082
