# Supplementary material for: QTL analysis of femaleness in monoecious spinach and fine mapping of a major QTL using an updated version of chromosome-scale pseudomolecules
Source: PLoS One. 2024 Feb 23;19(2):e0296675. doi: 10.1371/journal.pone.0296675 (PMC10890751; doi:10.1371/journal.pone.0296675)
Supplement: S8 Table — (PDF) [file pone.0296675.s021.pdf]

S8 Table. A linkage map and pseudomolecules of spinach.

Linkage Groups	Number of markers	Distance (cM)	Number of anchored scaffolds	SOL_r2.0_pseudomolecule	Length (bp)	Accession ID
LG1	424	89.1	7	Chr1	159,373,628	BTGF01000001
LG2	354	68.2	5	Chr2	136,918,686	BTGF01000002
LG3	287	106.6	16	Chr3	188,023,793	BTGF01000003
LG4	294	63.3	12	Chr4	126,785,950	BTGF01000004
LG5	238	62.2	13	Chr5	115,602,668	BTGF01000005
LG6	219	79.2	14	Chr6	152,476,739	BTGF01000006
Total	1816	468.6	65		879,181,464	
