# Supplementary material for: QTL analysis of femaleness in monoecious spinach and fine mapping of a major QTL using an updated version of chromosome-scale pseudomolecules
Source: PLoS One. 2024 Feb 23;19(2):e0296675. doi: 10.1371/journal.pone.0296675 (PMC10890751; doi:10.1371/journal.pone.0296675)
Supplement: S9 Table — (PDF) [file pone.0296675.s022.pdf]

S9 Table. ANOVA table showing the genetic interaction between loci qFem3.1/M and qFem2.1.

	df	Type III SS	LOD	%var	*F* value	*P* value
qFem3.1	6	5.8472	20.685	60.524	25.126	< 2e-16
qFem2.1	6	1.2202	6.421	12.631	5.243	0.000127
qFem2.1 : *qFem3.1*	4	0.4089	2.389	4.233	2.636	0.03971
