# Supplementary material for: QTL analysis of femaleness in monoecious spinach and fine mapping of a major QTL using an updated version of chromosome-scale pseudomolecules
Source: PLoS One. 2024 Feb 23;19(2):e0296675. doi: 10.1371/journal.pone.0296675 (PMC10890751; doi:10.1371/journal.pone.0296675)
Supplement: S10 Table — (PDF) [file pone.0296675.s023.pdf]

S10 Table. BP GO terms enriched in 136 up-regulated DEGs shared between the comparison pairs, 03-336 vs. 03-009 and NIL-M vs. 03-009.

GO. ID	GO Term	Annotated	Significant	Expected	*P* values from Fisher's exact test			
					classic	elim	weght	weght01
GO:0080167	response to karrikin	113	4	0.42	0.00082	0.00082	0.00082	0.00082
GO:0000966	RNA 5'-end processing	53	3	0.2	0.001	0.001	0.01122	0.00644
GO:0048455	stamen formation	14	2	0.05	0.00121	0.00121	0.00121	0.00121
GO:0007610	behavior	19	2	0.07	0.00224	0.00224	0.00224	0.00224
GO:0010093	specification of floral organ identity	26	2	0.1	0.00419	0.00419	0.00419	0.00419
GO:0010048	vernalization response	34	2	0.13	0.00709	0.00709	0.00709	0.00709
GO:0097237	cellular response to toxic substance	206	4	0.77	0.00717	0.00717	0.20041	0.07525
