# Supplementary material for: QTL analysis of femaleness in monoecious spinach and fine mapping of a major QTL using an updated version of chromosome-scale pseudomolecules
Source: PLoS One. 2024 Feb 23;19(2):e0296675. doi: 10.1371/journal.pone.0296675 (PMC10890751; doi:10.1371/journal.pone.0296675)
Supplement: S11 Table — (PDF) [file pone.0296675.s024.pdf]

S11 Table. BP GO terms enriched in 175 down-regulated-DEGs shared between the comparison pairs, 03-336 vs. 03-009 and NIL-M vs. 03-009.

					P values from Fisher's exact test			
GO. ID	GO Term	Annotated	Significant	Expected	classic	elim	weght	weght01
GO:0006298	mismatch repair	34	2	0.12	0.0062	0.0062	0.0062	0.0062
GO:0010025	wax biosynthetic process	42	2	0.15	0.0094	0.0094	0.0094	0.0094
GO:0090615	mitochondrial mRNA processing	43	2	0.15	0.0098	0.0098	0.0098	0.0098
