# Supplementary material for: QTL analysis of femaleness in monoecious spinach and fine mapping of a major QTL using an updated version of chromosome-scale pseudomolecules
Source: PLoS One. 2024 Feb 23;19(2):e0296675. doi: 10.1371/journal.pone.0296675 (PMC10890751; doi:10.1371/journal.pone.0296675)
Supplement: S12 Table — (PDF) [file pone.0296675.s025.pdf]

S12 Table. BP GO terms enriched in 836 up-regulated DEGs shared between the comparison pairs, 03-336 vs. 03-009 and 03-336 vs. NIL-M.

					P values from Fisher's exact test			
GO. ID	GO Term	Annotated	Significant	Expected	classic	elim	weght	weght01
GO:0050832	defense response to fungus	310	20	6.06	2.90E-06	2.90E-06	2.90E-06	3.60E-06
GO:0009554	megasporogenesis	17	5	0.33	1.40E-05	1.40E-05	1.40E-05	1.40E-05
GO:0048658	anther wall tapetum development	20	5	0.39	3.30E-05	3.30E-05	3.30E-05	3.30E-05
GO:0009556	microsporogenesis	29	5	0.57	0.00022	0.00022	0.00022	0.00022
GO:0010315	auxin export across the plasma membrane	44	5	0.86	0.00159	0.00159	0.00159	0.00159
GO:0006591	ornithine metabolic process	13	3	0.25	0.00182	0.00182	0.00182	0.00182
GO:0048455	stamen formation	14	3	0.27	0.00229	0.00229	0.00229	0.00229
GO:0010951	negative regulation of endopeptidase activity	29	4	0.57	0.0023	0.0023	0.0023	0.0023
GO:0010207	photosystem II assembly	17	3	0.33	0.00409	0.00409	0.00409	0.00409
GO:0006570	tyrosine metabolic process	19	3	0.37	0.00567	0.00567	0.00567	0.01243
GO:0010223	secondary shoot formation	40	4	0.78	0.00749	0.00749	0.00749	0.00749
GO:0009617	response to bacterium	533	19	10.41	0.00849	0.00849	0.02032	0.09383
