# Supplementary material for: QTL analysis of femaleness in monoecious spinach and fine mapping of a major QTL using an updated version of chromosome-scale pseudomolecules
Source: PLoS One. 2024 Feb 23;19(2):e0296675. doi: 10.1371/journal.pone.0296675 (PMC10890751; doi:10.1371/journal.pone.0296675)
Supplement: S13 Table — (PDF) [file pone.0296675.s026.pdf]

S13 Table. BP GO terms enriched in 1005 down-regulated DEGs shared between the comparison pairs, 03-336 vs. 03-009 and 03-336 vs. NIL-M.

GO. ID	GO Term	Annotated	Significant	Expected	*P* values from Fisher's exact test			
					classic	elim	weght	weght01
GO:0010048	vernalization response	34	6	0.75	8.60E-05	8.60E-05	8.60E-05	8.60E-05
GO:0098662	inorganic cation transmembrane transport	352	20	7.74	0.0001	0.0001	0.0001	0.0213
GO:0007163	establishment or maintenance of cell polarity	47	6	1.03	0.00054	0.00054	0.00054	0.0056
GO:0006952	defense response	1076	43	23.67	8.80E-05	0.00068	0.002	0.001
GO:0009909	regulation of flower development	213	13	4.69	0.00087	0.00087	1	0.002
GO:0009809	lignin biosynthetic process	53	6	1.17	0.00104	0.00104	0.00104	0.0141
GO:0031279	regulation of cyclase activity	11	3	0.24	0.00152	0.00152	0.00152	0.0015
GO:0035672	oligopeptide transmembrane transport	15	3	0.33	0.00394	0.00394	0.00394	0.0039
GO:0002188	translation reinitiation	16	3	0.35	0.00477	0.00477	0.00477	0.0048
GO:0048589	developmental growth	555	22	12.21	0.00558	0.00558	0.7996	1
GO:0006829	zinc ion transport	33	4	0.73	0.00568	0.00568	0.00568	0.0015
GO:0051262	protein tetramerization	35	4	0.77	0.00702	0.00702	0.00702	0.0522
GO:0030029	actin filament-based process	155	9	3.41	0.00731	0.00731	1	1
GO:2000243	positive regulation of reproductive process	78	6	1.72	0.00735	0.00735	0.00735	0.0695
GO:0098542	defense response to other organism	859	30	18.9	0.00802	0.00802	1	1
GO:0010380	regulation of chlorophyll biosynthetic process	37	4	0.81	0.00856	0.00856	0.00856	0.0086
