# Supplementary material for: QTL analysis of femaleness in monoecious spinach and fine mapping of a major QTL using an updated version of chromosome-scale pseudomolecules
Source: PLoS One. 2024 Feb 23;19(2):e0296675. doi: 10.1371/journal.pone.0296675 (PMC10890751; doi:10.1371/journal.pone.0296675)
Supplement: S14 Table — (PDF) [file pone.0296675.s027.pdf]

S14 Table. Candidate genes underlying QTLs for the monoecious condition.

				BLASTP				TPM			FDR *P*-value		
QTL	SOL_r1.1a gene id [16]	SOL_r2.0 gene id	Impact of variant	vs NR	E-value	vs Araport11	E-value	03-009	NIL-M	03-336	03-336 vs. 03-009	NIL-M vs. 03-339	03-336 vs. NIL-M
qFem2.1	g23600.t1	Chr2_g000510.1	HIGH	XP_021845944.1, pyruvate dehydrogenase E1 component subunit beta-1, mitochondrial-like isoform X1 [*S. oleracea*]	2.00E-208	AT5G50850.1, Transketolase family protein	0	12.47	13.79	11.15	0.846	1.000	0.648
	g23612.t1	Chr2_g000620.1	MODIFIER	XP_021845875.1, glutaredoxin-C5-like [*S. oleracea*]	0	AT5G14070.1, Thioredoxin superfamily protein	8.00E-53	35.73	33.48	92.74	0.006	1.000	0.002
	g23617.t1	Chr2_g000670.1	MODIFIER	XP_021845857.1, auxin-responsive protein SAUR32-like [*S. oleracea*]	1.60E-98	AT5G50760.1, SAUR-like auxin-responsive protein family	5.00E-22	0.56	0.12	3.34	0.174	0.918	0.036
	g23706.t3	Chr2_g001540.1	MODERATE	XP_021854904.1, pre-mRNA-splicing factor ATP-dependent RNA helicase DEAH7-like [*S. oleracea*]	0	AT5G13010.1, RNA helicase family protein	0	3.37	3.59	1.13	0.000	1.000	0.000
qFem6.1	g49564.t1	Chr6_g038890.1	MODERATE	XP_021866192.1 LRR receptor-like serine/threonine-protein kinase RPK2 [*S. oleracea*]	0	AT3G02130.2, receptor-like protein kinase 2	0	31.77	33.80	29.43	0.864	1.000	0.670
	g13001.t2	Chr6_g032140.1	MODERATE	XP_021851786.1 receptor protein kinase CLAVATA1-like [*S. oleracea*]	0	AT1G75820.1, Leucine-rich receptor-like protein kinase family protein	0	11.00	12.08	18.78	0.046	1.000	0.125
